# Household resilience and its role in sustaining food security in rural Bangladesh

**DOI:** 10.1371/journal.pone.0332868

**Published:** 2025-09-22

**Authors:** Ismat Tasnim, Md. Asif Iqbal, Ismat Ara Begum, Mohammad Jahangir Alam, Morten Graversgaard, Paresh Kumar Sarma, Kiril Manevski

**Affiliations:** 1 Department of Agribusiness and Marketing, Bangladesh Agricultural University, Mymensingh, Bangladesh; 2 Department of Agricultural Economics, Bangladesh Agricultural University, Mymensingh, Bangladesh; 3 Department of Agroecology, Aarhus University Interdisciplinary Center for Climate Change, Tjele, Denmark; 4 Bangladesh Agricultural University Research System, Bangladesh Agricultural University, Mymensingh, Bangladesh; Lusofona University of Humanities and Technologies: Universidade Lusofona de Humanidades e Tecnologias, PORTUGAL

## Abstract

Food insecurity and agriculture in South Asia, including Bangladesh, pose significant threats to the well-being and livelihoods of its people. Building adaptive capacities and resilient food systems is crucial for sustainable livelihoods. This study employs the Resilience Index Measurement and Analysis II framework to construct a Resilience Capacity Index (RCI) and analyze its relationship with food security using data from the Bangladesh Integrated Household Survey 2018. The study applies Exploratory Factor Analysis and Structural Equation Modeling to examine the impact of key resilience components such as Access to Basic Services, Adaptive Capacity, and Assets on household resilience. The findings reveal that access to basic services, land assets, and farm equipment positively influences households’ resilience capacity. However, the presence of livestock assets has a negative impact, potentially due to market volatility, climate vulnerability, and disease outbreaks. Additionally, adaptive capacity has a positive but insignificant influence on RCI, suggesting that without enhancing economic opportunities, institutional support, and inclusive development strategies, adaptive capacity could not be enough to foster resilience. However, resilient capacity enhances food security metrics such as the Food Consumption Score and Expenditure. These findings underscore the importance of policies that focus on increasing and maintaining access to basic services, promoting sustainable land management practices, and strengthening social safety nets. This study emphasizes the importance of focusing on livestock assets to ensure their sustainability by stabilizing the livestock market, improving veterinary services, and providing subsidies to reduce maintenance costs.

## 1. Introduction

Food consumption is a fundamental human need, and a global concern, characterized by a surplus of food in some regions and scarcity in others. It stands as a cornerstone of the United Nations’ Sustainable Development Goals (SDGs), notably SDG-1 (No Hunger) [1]. Achieving food security, which ensures an end to hunger, is essential for human well-being [2]. According to the World Food Summit, food security entails access to sufficient, safe, and nutritious food [3]. However, food security is a multifaceted issue intertwined with trade, politics, history, health and nutrition [4]. Countries reliant on imports are often vulnerable to fluctuations in the global market, as international trade can directly affect food accessibility and price stability [5,6]. Political instability and conflict can severely disrupt food production and distribution systems, as observed in countries like Somalia and Yemen [7]. Food systems and access continue to be shaped by historical legacies of colonialism and unequal land distribution, particularly in many developing countries [8]. Moreover, malnutrition exacerbates health vulnerabilities, while poor health undermines households’ capacity to produce or procure food, creating a cyclical relationship [9,10]. Achieving food security, therefore, requires nutrition-sensitive systems that go beyond meeting calorie needs to ensure dietary diversity and adequate nutrient intake [11,12]. Food insecurity, on the other hand, refers to limited or uncertain access to adequate and nutritious food, and the inability to obtain food in socially acceptable ways [13]. It represents a complex challenge that affects individuals and communities worldwide.

Many developing countries, including Bangladesh, have recently experienced acute food insecurity. According to the Integrated Food Security Phase Classification (IPC), a collaborative initiative involving governments, non-governmental organizations, UN Agencies, civil society and other relevant actors, approximately 9.14 million individuals, or 20% of the population, are categorized in Phases 3 and 4 [14]. These phases indicate high levels of acute food insecurity in certain parts of Bangladesh. The poorest households in these areas face significant challenges exacerbated by factors such as high inflation, reduced earnings, and frequent climate-related shocks, which contribute to the severity of food insecurity among the population [14].

The frequency and intensity of natural, economic, and political hazards affecting individuals, businesses, economies, and even entire nations are increasing [15–19]. The concept of resilience has thus gained prominence in governmental and research contexts. Initially introduced in ecology literature by Holling [20], resilience has since been studied and applied across various fields, including socioeconomics [21]. Resilience is commonly defined as the capacity to anticipate, prepare for, respond to, and recover from shocks efficiently and effectively [22]. Recent studies have expanded this definition to include the ability to manage persistent risk exposure [23]. In the context of food security analysis, resilience refers to the latent capacity of households to withstand and navigate socioeconomic factors, climate variability, and other shocks [24]. Traditionally viewed as an endpoint, resilience is now understood as a continuous process that can either enhance or diminish response capacities, thereby impacting overall well-being [25].

Resilient food systems are adept at adapting to new conditions while maintaining stable food production and ensuring access for vulnerable populations. They mitigate the impact of shocks and stressors, with social and economic factors playing crucial roles in enhancing resilience. In countries like Bangladesh, where a significant portion of the population relies on agriculture, food insecurity remains a pressing concern. The food system is vulnerable due to its heavy reliance on a few staple crops such as rice, wheat, and maize, and its interconnectedness with the global food supply chain [14,26–28]. Disruptions in one part of the system can lead to cascading effects throughout. Moreover, the lack of agricultural diversity and reliance on intensive monoculture practices increase vulnerability to pests, diseases, and climate shocks. Bangladesh is particularly susceptible to extreme weather events such as heat waves, floods and droughts [29], which can severely impact food production and access [30].

Assessing the resilience of communities and households to shocks and threats is crucial as it allows nations to anticipate and effectively address these challenges. Resilience assessments help policymakers and organisations understand how well a community can rebound from adversity. Food security, with its multifaceted and complex nature, has predominantly been studied through vulnerability assessments. However, the methodologies used in these assessments often lack dynamism, which makes precise predictions of potential shocks and incidents challenging [31–33].

The Food and Agriculture Organization (FAO) developed the Resilience Index Measurement Analysis (RIMA) to quantify resilience [6,34,35]. This method introduces the concept that resilience can be estimated as a latent variable through a two-step procedure involving observable factors, even when resilience itself is not immediately observable [33]. Recent advancements by FAO have enhanced this approach by using structural equation modelling instead of factor analysis to estimate the resilience index. Additionally, more variables have been included as proxies to represent institutional and natural environments [36].

Applying the RIMA-II model in Bangladesh offers a novel, structured econometric approach to measuring household resilience, an aspect largely absent in previous food security studies. Unlike traditional vulnerability assessments that rely on static indicators, RIMA-II conceptualizes resilience as a latent variable, allowing for a more nuanced and dynamic analysis of the factors influencing resilience capacity. While RIMA-II has been applied in various regions, its use in Bangladesh remains limited, with most existing studies focusing on food security determinants [37,38] or coping strategies [39–48] rather than the interactions among resilience components. Furthermore, prior research lacks a comprehensive resilience index beyond traditional vulnerability assessments, limiting its ability to capture the complex interconnections among resilience factors. Thus, this study makes two main contributions. Firstly, it investigates the key determinants shaping the resilience capacity of households, which influence their ability to adapt and recover from adverse conditions. Second, it assesses how resilience capacity translates into improved food security outcomes. By situating this analysis within the broader context of rural livelihoods, the study aims to provide empirical insights into the mechanisms through which resilience serves as a buffer against food insecurity, offering implications for policy interventions aimed at fostering sustainable rural development.

## 2. Review of literature

When attempting to understand and enhance resilience, it is crucial to provide necessary resources and techniques to mitigate adverse outcomes, predict and prepare for shocks and stress, eliminate destructive coping mechanisms, and facilitate recovery [49]. Resilience in environmental and socioeconomic systems is demonstrated by responses to negative events and shocks [21], which encompass transformation, adaptation, and absorption responses [50–53]. Resilience, viewed as an intermediate outcome in socioeconomic studies, integrates capacities such as a household’s or community’s ability to cope with shocks [53]. Recent studies have endeavoured to integrate resilience into various development contexts, including food safety [22,54], well-being [55,56], rural development [57–59], and sustainable livelihoods [60–62].

The articles by [32,33] represent early efforts to define and quantify household resilience in the context of food insecurity. These studies focused on the household as the primary unit of analysis, recognising that crucial decisions regarding risk management made at this level, both before and after adverse events impacting food security [33] introduced a resilience index, treated as a latent variable that cannot be directly observed, and employed a two-stage factor analysis approach using measurable variables. Building on this framework [63] applied a similar latent variable model to measure resilience in Northern Bangladesh, highlighting the importance of comprehensively understanding resilience factors across various capacities. Additionally, d’Errico et al. investigated how shocks, particularly conflicts and climate events, influence resilience measurement [24].

Similarly, Ciani et al. aimed to assess household resilience to food insecurity in a changing environment using principal components and multivariate analysis of panel data. Their resilience index indicated that small landowners and agricultural wage workers exhibited lower resilience levels compared to other livelihood groups [64]. Dhraief et al. conducted a cross-sectional survey employing factor analysis and regression models. Their findings highlighted the significant roles of income and food access, adaptive capacity, and social safety nets in enhancing household resilience, as evidenced by their positive correlations with the resilience index [65]. Conversely, asset possession and climate change showed negative associations with household resilience in their study. However, contrasting findings in other studies have indicated that assets can have positive and notable effects [49,66,67].

Rural households generally exhibit less resilient to food shortages compared to urban and peri-urban households [68]. However, during the COVID-19 lockdowns, urban households experienced higher levels of food insecurity than rural households, yet they were more likely to resort to financial and food-compromising coping strategies [48]. Sassi et al. argued that households often turn to less preferred and cheaper food options during times of food insecurity, which can lead to long-term societal impacts and reduced ability to cope [69]. Large family households are particularly vulnerable to food insecurity, often relying on lower-quality meals and support from family or friends [70]. Ownership of assets also plays a crucial role in the resilience of smallholder farmers, with households possessing more assets generally exhibiting greater resilience to food insecurity [66]. Food-secure households typically exhibit higher dietary diversity and rely less on coping mechanisms, benefitting from adaptive and absorptive resilience capacities along with sufficient income [71].

Previous studies have highlighted that household resilience to food insecurity is influenced by factors such as income, assets, social safety nets, and adaptive capacity, with resilience levels varying across different livelihood groups and geographic locations [32,34,72,73]. However, findings on the role of assets remain inconclusive, calling for a more nuanced approach that differentiates asset types to better capture their distinct contributions to resilience. Despite the growing use of resilience measurement frameworks, studies in Bangladesh have yet to adopt a comprehensive approach that captures the interplay between various resilience components. Although prior research has identified the resilience determinants [48,63,74], few studies have specifically examined the suitability of RIMA-II for the Bangladeshi context [75]. Traditional vulnerability assessments often fail to account for critical dimensions such as access to services, adaptive strategies, and the differential roles of asset types as drivers of resilience. This study addresses these gaps by employing RIMA-II to systematically measure resilience as a latent construct, offering a more integrated and nuanced approach compared to earlier methods in rural Bangladesh. A key contribution of this research is the disaggregation of asset components, land, livestock, and farm equipment, to assess their distinct effects on resilience. This disaggregation is essential, as each asset type contributes differently to resilience in rural Bangladesh. Land ownership is vital for food production and long-term economic stability [76–78], while livestock assets, despite their perceived benefits, introduce risks related to market volatility [79–81], disease outbreaks [82–88], and climate vulnerability [89–92]. Farm equipment, on the other hand, plays a key role in mechanization and agricultural efficiency, making it distinct from the other asset categories [93–97]. Treating assets as a single, aggregate variable would overlook these critical differences in how they contribute to or detract from resilience. This approach ensures a more accurate and policy-relevant analysis of household resilience dynamics in Bangladesh. Furthermore, the use of Structural Equation Modeling (SEM) to validate resilience determinants has been rare in prior research, emphasizing the need for this methodological advancement.

By combining RIMA-II with a robust empirical framework, this study advances the understanding of resilience pathways and generates policy-relevant insights for improving food security in Bangladesh.

## 3. Theoretical discussion and hypothesis formulation

### 3.1. RIMA II Model

It is increasingly recognised that one of the most effective ways to reduce or even avert food security crises is to create more resilient livelihoods [24,49]. The Resilience Index Measurement and Analysis (RIMA) was developed within this framework, with pioneering contributions by the FAO [34]. Employing a novel quantitative methodology, RIMA helps explain why and how certain households can withstand shocks and stressors more effectively than others. Resilience, being a multidimensional concept that cannot be directly observed, must be assessed through proxies by focusing on the needs of the most vulnerable groups. The new RIMA-II methodology offers enhanced guidance for successfully planning, implementing, overseeing, and assessing aid. The RIMA II model was employed to identify and study household resilience [36].

The RIMA-II model enables a better understanding of households` circumstances and needs, as well as identifying potential areas for improving future assistance. Access to Basic Services (ABS), Assets (AST), and Adaptive Capacity (AC) are the widely recognised pillars of the Resilience Capacity Index (RCI). Due to the lack of available data on Social Safety Nets (SSNs), our study focuses on the other three pillars. To estimate each pillar, variables were considered based on a literature review, data availability, context analysis, and statistical properties.

ABS refers to the ability to meet households’ basic needs and encompasses the quality, effectiveness, and access to basic services [98]. This study assesses ABS through the following components: floor type (X1), wall type (X2), housing condition (X3), roof type (X4), sanitation type (X5), source of water (X6), cooking fuel type (X7), distance to health facilities from residence (in kilometers) (X8) and distance to the market (in kilometers) (X9).

AC refers to a household’s ability to cope with and adapt to an unfamiliar situation, enabling them to maintain everyday affairs without long-term disruptions [32,36]. It indicates that more adaptive residents can handle adverse situations without reducing their standard of living [49,99]. In this study, this pillar includes the following components: household head`s employment location (X10), household head`s occupation type (X11), savings (X12), household income per month (X13), household head`s education year (X14), and diversified sources of income (X15).

An essential prerequisite for shock response is AST, the second pillar of the RIMA-II model [32]. Assets encompass a household’s income-producing and not-producing assets [98], serving as indicators of the household’s economic circumstances and providing insight into the impact of shocks on households. The proxies used to construct AST include land holding (in decimal) (X16), land value (X17), livestock value (X18), livestock quantity (X19), farm equipment value (X20), farm equipment quantity (X21), and non-agricultural asset value (X22).

The RIMA-II is divided into two sections: a descriptive and a causal analysis [98]. The RCI used to assess households and inform policy, is generated through descriptive analysis. The RCI is constructed in two stages. The first stage, the formative model, employs factor analysis of observable variables to estimate attributes (pillars) of resilience. The second stage, the measurement model, utilizes a Multiple Indicators Multiple Causes (MIMIC) model to calculate the RCI based on these pillars while considering the relationships between RCI and food security indicators. The developmental model links resilience to the pillars, while the measurement model focuses on food security indicators such as the Food Consumption Score (FCS) and Food Expenditure (FX).

**Food Consumption Score (FCS)** collects household-level data on the diversity and frequency of food groups consumed over seven days. This data is weighted according to the relative nutritional value of each food group. The main food groups and their respective weights are: staples (weight 2), pulses (weight 3), vegetables (weight 1), fruit (weight 1), meat/fish (weight 4), milk (weight 4), sugar (weight 0.5) and oil (weight 0.5). To calculate FCS, the first step is to sum the consumption frequencies of food items within each group. Next, each food group`s total is multiplied by its respective weight [100].

**Food Expenditure (FX)** refers to the total cost of food obtained, whether purchased or acquired through other means, including both alcoholic and non-alcoholic beverages. This calculation also encompasses food expenditures on dining out at venues such as bars, restaurants, food courts, workplace canteens and street vendors [101]. FX is calculated by multiplying the quantity of food consumed over a seven-day period by its unit price, expressed in Bangladeshi Tk (1 US$ = 110 BDT (Bangladesh Bank, 2024).

### 3.2. Formulation of Hypotheses

**Fig 1:**
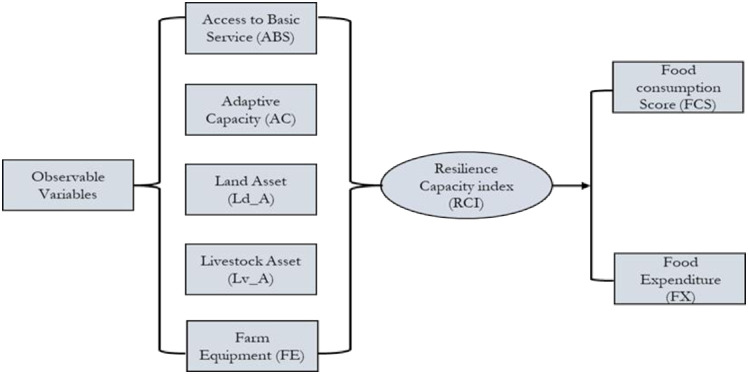
Hypothetical framework.

Based on the literature and the conceptual framework depicted in Fig 1, observable variables are expected to significantly influence latent variables or pillars. Furthermore, these latent variables are expected to have a significant impact on the RCI. Finally, the RCI is expected to significantly influence food security indicators, specifically, FCS and FX. Previous studies [14,15,19,20,25,29,41–43,76–79] have consistently highlighted the substantial influence of various observable variables on latent pillars, the significant impact of latent pillars on RCI, and the consequential effect of RCI on food security. Consequently, this study proposes the following hypotheses:

Hypothesis A. Access to Basic Services (ABS) significantly and positively influences the RCI.
Hypothesis B. Adaptive Capacity (AC) significantly and positively influences the RCI.
Hypothesis C. Land Asset (Ld_A) significantly and positively influences the RCI.
Hypothesis D. Livestock Asset (Lv_A) significantly and positively influences the RCI.
Hypothesis E. Farm Equipment (FE) significantly and positively influences the RCI.
Hypothesis F. The RCI significantly and positively influences the food consumption score (FCS).
Hypothesis G. The RCI significantly and positively influences food expenditure (FX).

## 4. Materials & methods

### 4.1. Data source

The observed variables (X1, X2,.. Xn) used in this study were derived from the Bangladesh Integrated Household Survey (BIHS) 2018. This household survey is administered by Data Analysis and Technical Assistance Limited and designed and supervised by the International Food Policy Research Institute (IFPRI). The BIHS sample is statistically representative of rural Bangladesh, covering the nation`s seven administrative divisions: Barisal, Chittagong, Dhaka, Khulna, Rajshahi, Rangpur, and Sylhet. The survey includes 325 primary sampling units (PSUs) or villages, encompassing a total of 5,604 households in the BIHS dataset. Therefore, this study utilises a cross-sectional dataset of 5,604 households to achieve its research objectives. This dataset serves as the basis for assessing resilience through the pillars of ABS, AC, and AST, and their impact on the RCI and food security indicators.

### 4.2. Measures

Twenty-two measurement items were used to construct latent pillars of the RCI, which is presented in Appendix Table 1 in S1 File. Each observable variable was assessed using a five-point summated Likert scale, where respondents rated their conditions (1 = very poor; 2 = poor; 3 = moderate; 4 = rich; 5 = very rich).

### 4.3. Data analysis

The study utilized a quantitative approach. Initially, descriptive statistics were used to summarize and describe the observable variables of the pillars related to the pillars of resilience and food security indicators. This involved calculating measures such as means, standard deviations, and frequency distributions to provide a clear picture of the characteristics of the data. Statistical factorial analyses were employed to test the study’s hypotheses. To assess the factorial validity of the constructs and explore the latent pillars in the data, exploratory factor analysis was performed following [102]. Given the interconnections among latent constructs, we allowed for inter-factor correlation using the maximum likelihood factor extraction method with orthogonal varimax rotation. Subsequently, structural equation modeling (SEM) was exclusively used for descriptive purposes to understand the explanatory power of the pillars and their relationships with the RCI. This approach is a powerful statistical technique that allows researchers to test complex relationships among variables. It was used in this study to examine the linear correlations between the underlying factors (in this case, the pillars) and the latent variable RCI [103].

The formative model, represented by structural Equation (1), links resilience (η) to the pillars (xi). In contrast, the measurement model, represented by Equation (2), is defined by the Food Consumption Score (FCS) and household food expenditure (FX).


$$\;[\eta =\;[\beta_{\text{1}},\;\beta_{\text{2}},\;\beta_{\text{3}},\;\beta_{\text{4}},\;\beta_{\text{5}}]\;\times [\begin{array}{c} ABS\\ \begin{array}{c} AC\\ \begin{array}{c} Ld\_A\\ \begin{array}{c} Lv\_A\\ FE \end{array} \end{array} \end{array} \end{array}]\;+\;[\varepsilon_{\text{1}}]$$ (1)

$$\left[\begin{array}{c} FX\\ FCS \end{array}]\;=\;[\wedge_{\text{1}},\;\wedge_{\text{2}}]\;\times \;[\eta \;=\;RCI]\;+\;[\varepsilon_{FX},\;\varepsilon_{FCS}\right]$$ (2)

**Fig 2:**
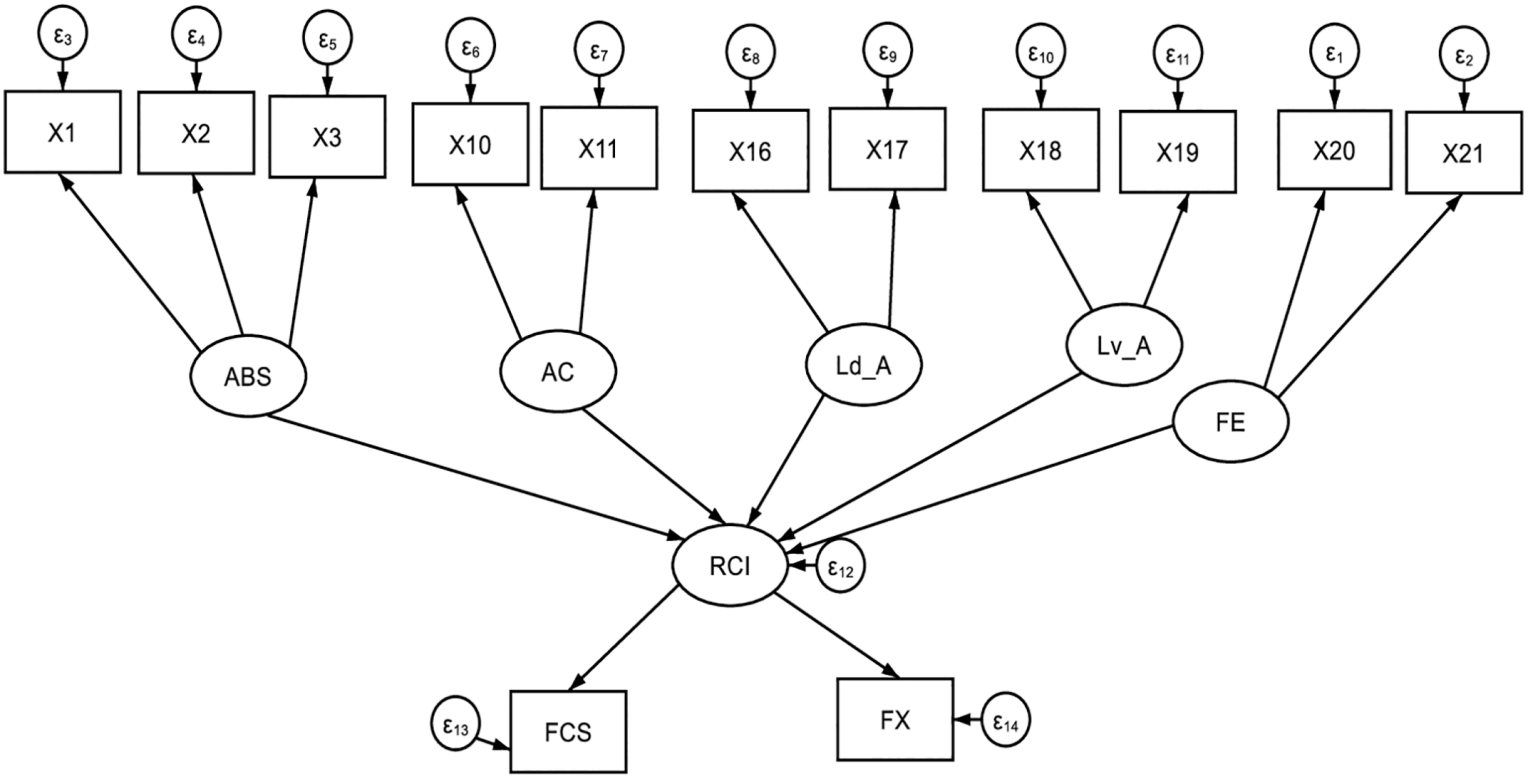
Structural equation model.


The upper portion of the path diagram (Fig 2) illustrates the relationship between the resilience pillars, as measured by error ε_R_, and the disturbance term of the structural estimation. The measuring model is represented by arrows pointing toward the food consumption Score and the food expenditure in the lower half of the structural model. Since the estimated RCI lacks a specific measurement scale, a scale has been proposed with the coefficient of the food consumption loading (˄_1_) set to 1. This assumption posits that a one standard deviation increase in RCI corresponds to a one standard deviation increase in food intake. This determination also establishes the unit of measurement for the other outcome indicator (˄_2_), alongside the variance of the two food security indicators, as depicted in Equations (3) and (4):

$$FX\;=\;\wedge_{\text{1}}\;RCI\;+\;\varepsilon_{FX}$$ (3)

$$FCS\;=\;\wedge_{\text{2}}\;RCI\;+\;\varepsilon_{FCS}$$ (4)

### 4.4. Ethical approval

The study was conducted in accordance with the Declaration of Helsinki. It utilized publicly available data from the Bangladesh Integrated Household Survey (BIHS), administered by the International Food Policy Research Institute (IFPRI). The BIHS obtained formal written informed consent from all respondents prior to data collection.

## 5. Result & discussion

### 5.1. Descriptive statistics

The summary statistics of all the attributes used to construct the resilience pillars are presented in Appendix Table 2 in S1 File. According to the established criteria [102,104], data is considered normal if kurtosis falls between −7 and +7 and the skewness between −2 and +2. However, our results indicate that some attribute values exceed these thresholds, suggesting potential outliers that may be excluded from further analysis.

### 5.2. Scale reliability & identifying latent variables through exploratory factor analysis

Cronbach’s alpha assesses with which respondents answer items on a scale or subscale [105]. A Cronbach’s alpha score greater than 0.6 is generally considered reliable with values between 0.6 to 0.8 deemed acceptable [106,107]. The reliability alpha value for the adaptive capacity (AC) pillar significantly exceeded the threshold of 0.70, indicating adequate reliability [105]. The other four pillars also demonstrated reliability, with Cronbach’s alpha values greater than 0.60 [106–108].

Following the initial reliability assessment, Exploratory Factor Analysis (EFA) was employed to explore the latent factor structure. The maximum likelihood method was used to identify components and establish the pattern matrix. The Kaiser-Meyer-Olkin (KMO) measure of sampling adequacy was 0.778, surpassing the maximum acceptable level of 0.5 [109]. The criteria, which include a significant correlation matrix determinant of 0.009, a KMO value of 0.778, and a significant Bartlett’s Chi-square of χ2 = 26648.163, p = 0.000, confirmed the adequacy of the sample size chosen for the study [110], indicating sufficient intercorrelation for factorial analysis. Initially identifying ten factors, we retained five factors that collectively explained 82.68% of the total variance in the data, exceeding the acceptable threshold of 60% [102]. Table 1 presents the pattern matrix, demonstrating how each numbered questionnaire item loaded precisely (values above 0.50 on a single component) onto its respective factor. Typically, minimum item-factor loadings of 0.3 to 0.4 are recommended [111–113].

**Table 1:** Results of exploratory factor analysis.

Variable	Factor1	Factor2	Factor3	Factor4	Factor5
X1	0.642				
X2	0.624				
X3	0.604				
X10		0.859			
X11		0.803			
X16			0.820		
X17			0.669		
X18				0.922	
X19				0.610	
X20					0.860
X21					0.513
Variance explained	29.74	23.49	11.09	10.25	8.11
Eigenvalue	2.98	2.35	1.11	1.03	0.81
Cronbach’s α	0.6690	0.8236	0.7544	0.7538	0.6961

Table 1 categorizes items X1(floor type), X2 (wall type), and X3 (housing condition) under factor 1 as ABS, explaining 29.74% of the total variance. Factor 2, labelled AC, includes X10 (household head`s employment location) and X11 (household head`s occupation type), explaining 23.49% of the variance. Factors 3 and 4 are labelled Ld_A and Lv_A, respectively, and explain 11.09% and 10.25% of the variance. They comprise items X16 (land size), X17 (land value), X18 (livestock value), and X19 (livestock quantity). Item X20 (farm equipment value) and X21 (farm equipment quantity) of factor 5 are labelled FE. Although factors Ld_A, Lv_A, and FE collectively represent the single factor named Assets (AST), they are labelled as separate factors based on our country’s perspective. The loadings of the items range from 0.51 to 0.92.

Table 2 presents the correlation matrix of the five factors included in the analysis. ABS shows significant positive correlations with Ld_A (0.042) and FE (0.034) at the 1% significance level, as these factors contribute to higher income levels that enable households to afford better services. ABS also negatively correlates with AC (0.028) and Lv_A (0.026) at the 5% and 10% significance levels, respectively, possibly due to market volatility and health-related concerns affecting livestock income and service accessibility. AC demonstrates a significant positive correlation with Ld_A (0.023) and Lv_A (0.035) at the 10% and 1% significance levels, respectively, but shows an insignificant correlation with FE (0.017). Ld_A shows a significant positive correlation with both Lv_A (0.047) and FE (0.072) at the 1% level. Lv_A and FE correlate significantly at the 5% level (0.030). Despite the correlations observed among factors, there is no evidence of significant multicollinearity, as no correlation coefficient exceeds 0.8 [114–116].

**Table 2:** Correlation matrix between factors.

Factors	ABS	AC	Ld_A	Lv_A	FE
ABS	1.000				
AC	−0.028**	1.000			
Ld_A	0.042***	0.023*	1.000		
Lv_A	−0.026*	0.035***	0.047***	1.000	
FE	0.034***	0.017	0.072***	0.030**	1.000

Note: ***p < 0.01, **p < 0.05 and *p < 0.10.

### 5.3. Structural equation model (SEM) and hypotheses test

Table 3 presents the findings from the multiple indicators multiple causes (MIMIC) model, which was derived using structural equation modelling. In this case, we consider the broader contextual framework to accurately analyze the 2018 data, which includes ongoing food insecurity, a substantial influx of Rohingya refugees facing acute malnutrition amidst monsoon-related hazards, and deep-rooted structural vulnerabilities such as limited agricultural diversification. These factors influenced both households` potential for resilience and the outcomes observed in our SEM model. Study shows that ABS exhibits a significant coefficient of 0.429, indicating that a well-structured living condition with essential facilities enhances households’ resilience capacity. This finding also indicates that households with better access to essential services are more equipped to cope with food insecurity and recover from shocks. Thus, the study’s findings support hypothesis A: access to basic services significantly influences the resilience capacity index. Our results reveal a positive correlation between ABS and RCI (Table 3), consistent with previous studies [32,36,117,118], which suggest that access to basic services enhances economic opportunities, knowledge, and overall well-being, thereby contributing to greater resilience. In contrast, studies by [53,66,119,120] report a negative association, attributing decreasing service quality as households become poorer in their respective countries. Previous studies have often identified AC as a significant influencing factor [24,65,67,121,117]. However, our findings indicate that AC is a positive but insignificant factor, suggesting that institutional and economic barriers impede the effectiveness of this factor. While education, skill development, access to information, and access to credit are critical components of AC, their impact on resilience is highly dependent on external factors such as labor market conditions and institutional access [122,123]. This discrepancy may be attributed to Bangladesh`s high vulnerability to global changes, significant socio-economic challenges, and limitations in institutional capacity [124–126]. In Bangladesh, people are unable to leverage their adaptive skills for resilience due to a lack of employment opportunities [119,125,127,128]. Moreover, limited access to credit, and financial services may restrict households from transferring their adaptive strategies to long-term stability [43,125,128]. Furthermore, gender-based disparities in access to resources and decision-making power also undermine the significance of AC [54,129–133]. As a result, households in Bangladesh may exhibit lower adaptive capacity compared to those in other developing or developed countries [43,47,134,135]. Thus, our results do not support hypothesis B: adaptive capacity significantly influences the resilience capacity index. The “Resilience Index Measurement and Analysis II” report by the Food and Agriculture Organization (FAO) discusses how adaptive capacity contributes to resilience but notes that its impact may not always be significant in certain contexts [36].

**Table 3:** Multiple Indicators Multiple Causes (MIMIC) model of RCI.

Structural Component	ABS		0.429*** (0.020)
	AC		0.008 (0.019)
	Ld_A		0.119*** (0.019)
	Lv_A		−0.053** (0.022)
	FE		0.042* (0.026)
Measurement Component	Food Consumption Score	RCI	0.778*** (0.019)
		_cons	2.909*** (0.030)
	Food Expenditure	RCI	0.688*** (0.017)
		_cons	2.605*** (0.028)
Observations	N		5,604

Note: Standard errors in parentheses, ***p < 0.01.

The critical role of assets in resilience capacity has been widely recognised, as they constitute the most significant pillar for the RCI. Similar findings have been reported by other authors, such as [49,66,67,119] in the context of Malian and Ethiopian households, highlighting assets as the most pertinent factor for RCI, which aligns with our study findings. However, this study takes a more granular approach to segregating the assets into three distinct categories; land asset, livestock asset, and farm equipment, to identify their unique contributions to resilience building. This disaggregation is crucial, as each asset type plays a unique role in shaping a household`s ability to withstand and recover from food insecurity shocks. In the context of Bangladesh, only a small proportion of households own all three asset types of assets simultaneously, making it essential to analyse their individual and combined impacts on resilience. Our results demonstrate a significant coefficient of 0.119 for Ld_A and 0.042 for FE, indicating a positive influence on RCI, thereby corroborating hypotheses C and E. Land assets are crucial for resilience building as they provide financial stability and income through agriculture [76–78,136]. Additionally, having farm equipment ensures household mechanization and agricultural efficiency, which boosts farm production and enhances household resilience [93–97]. Since these assets play distinct roles in shaping resilience capacity, it is essential to calculate them separately.

However, Lv_A demonstrates a significant negative value of 0.053 suggesting that it has a significant negative impact on resilience in Bangladesh. Because it has a high mortality rate of livestock due to natural disasters and high disease outbreaks [137,138]. High vulnerability to climate change may, directly and indirectly, affect livestock production systems, resulting in negative asset values [47,89–92,139–142]. Lv_A are influenced by several interrelated factors that exhibit a counterintuitive negative relationship with resilience, such as market volatility, disease outbreaks, high maintenance cost, and climatic vulnerability. During economic downturns or supply chain disruptions, farmers are forced to sell their livestock at lower prices, undermining their financial stability [79–81]. Disease outbreaks, such as foot-and-mouth disease or avian influenza, frequently affect livestock in Bangladesh, resulting in significant economic losses for rural households. Limited access to veterinary care and inadequate disease prevention measures exacerbate this issue [82–88]. Livestock also involves costly sectors such as feeding, healthcare, and shelter, imposing additional financial burdens on households. While land or farm equipment offers long-term productivity gains, livestock requires continuous investment, making it a riskier asset for resilience building. These combined factors suggest that although livestock is traditionally seen as a liquid asset, it needs more infrastructural development to contribute to long-term resilience in the Bangladeshi context. Consequently, Lv_A cannot verify hypothesis D, which suggests that livestock assets positively influence resilience capacity. Our study indicates that RCI positively influences FCS and FX with significant coefficients of 0.778 and 0.688, respectively. That means households with higher resilience are less likely to experience deteriorating food security and are better positioned to maintain or restore food consumption and expenditure [24]. Policymakers should prioritize resilience-building as a core component of food security programming, employing multidimensional frameworks such as RIMA-II in Bangladesh for targeted, long-term strategies. Our results support hypotheses F and G, which concern the impact of resilience on food consumption and expenditure.

These outcomes remain robust across various model specifications. The results presented in Table 4 indicate that the model under consideration exhibits a significant likelihood ratio Chi-square of 1200.82 at the 1% significance level. The first chi-square compares the model against the saturated test, while the second chi-square compares the baseline versus the saturated model. The saturated model demonstrates a perfect fit for the covariance structure. According to Browne et al., the root mean squared error of approximation (RMSEA) indicates an adequate fit with values between 0.05 and 0.08 [143]. A value close to one on both the Tucker-Lewis index (TLI) and the comparative fit index (CFI) denotes a good fit, as suggested by Hair et al. and Bentler [102,144]. The coefficient of determination (CD = 1.00) also confirms a perfect fit. Typically, for an adequate fit, the standardised root mean square residual (SRMR) should be less than 0.05, as recommended by Hu and Bentler [145].

**Table 4:** Goodness of fit.

Fit statistic	Value	Description
Likelihood ratio		
χ2 _ms (50)	1200.82	model vs. saturated
p > chi2	0.00	
χ2_bs (72)	20613.84	baseline vs. saturated
p > chi2	0.00	
Population error		
RMSEA	0.064	Root mean squared error of approximation
P close	0.000	Probability RMSEA ≤ 0.05
Baseline comparison		
CFI	0.944	Comparative fit index
TLI	0.913	Tucker-Lewis index
Size of residuals		
CD	1.000	Coefficient of determination
SRMR	0.047	Standardized root mean squared residual

## 6. Conclusion & recommendations

Our study results demonstrate that access to basic services, land assets, and farm equipment significantly enhances household resilience. The positive impact of these factors on RCI suggests promising avenues for further research. Future studies could delve into how these components bolster agricultural system resilience, aiding government efforts in designing effective action plans. Exploring potential interactions among these factors could yield insights into the intricate dynamics of resilience. Interestingly, adaptive capacity showed no noticeable impact on RCI, highlighting the distinction between adaptation and resilience. Although our study indicates that livestock has a negative impact on resilience, it remains an invaluable resource for food security, income generation, and financial stability in rural economies. Livestock plays a crucial role in resilience management by acting as a liquid asset during economic crises, enhancing agricultural output through soil fertility and draft power, and improving access to financing.

Building resilience remains the most effective strategy to withstand shocks. In developing countries, policies and programs aimed at improving household resilience can significantly advance long-term food security. Humanitarian resilience initiatives should prioritize addressing rural populations` needs, as they face heightened exposure to food insecurity. Study results indicate that prioritising access to basic services such as healthcare, education, and sanitation in rural areas is crucial for fostering local and national resilience. Furthermore, enhancing incentives for land protection and promoting sustainable land management practices can enhance agricultural resilience. Policies supporting agricultural technology and equipment can also bolster resilience. Implementing social safety nets and encouraging farmers to diversify their revenue streams are effective strategies beyond human-centric protection measures. To enhance the sustainability of livestock assets, policymakers should focus on improving the affordability and accessibility of veterinary services and climate-resilient livestock management at the grassroots level. Subsidies on livestock medicine, feed, and improved breeds should also be provided to reduce maintenance costs. Strengthening adaptive capacity requires skill-based education, financial inclusion, and institutional support to foster entrepreneurship and human capital development, thereby creating more employment opportunities and improving infrastructure. Additionally, targeted awareness programs should encourage women’s participation in household and community decision-making, ensuring gender-inclusive resilience-building strategies.

However, the data used in our analysis were not explicitly gathered for resilience measurement purposes, which may limit the depth and accuracy of our study. Using cross-sectional data limits the ability to capture dynamic changes in household resilience over time, as it provides only a snapshot rather than reflecting long-term trends. Thereby restricts our ability to draw definitive conclusions about long-term resilience to food insecurity. It is important to acknowledge that, while this study emphasizes the role of land access and basic services in strengthening household resilience, social capital, gender dynamics, and power disparities are also significant, though less visible, factors influencing resilience in rural Bangladesh. These factors could not be explicitly incorporated into the SEM analysis due to dataset constraints. Furthermore, the absence of data related to recent shocks, disasters, or conflicts in our analysis limits the comprehensiveness of our study. These data constraints hinder our ability to examine the impact of acute events on household resilience, potentially overlooking critical dimensions of resilience dynamics. Future research should utilize longitudinal datasets specifically designed for resilience measurement, incorporating multiple survey rounds to capture temporal dynamics. Additionally, integrating socio-institutional variables with real-time data on shocks, disasters, and conflicts will enhance the comprehensiveness of resilience analysis, enabling a more accurate assessment of household responses to food insecurity.

## Supporting information

S1 File **Appendix Table 1**. Description of the variables. **Appendix Table 2**. Descriptive statistics.
(DOCX)
