# Supplementary material for: Household resilience and its role in sustaining food security in rural Bangladesh
Source: PLoS One. 2025 Sep 22;20(9):e0332868. doi: 10.1371/journal.pone.0332868 (PMC12453207; doi:10.1371/journal.pone.0332868)
Supplement: S1 File — Appendix Table 1. Description of the variables. Appendix Table 2. Descriptive statistics. (DOCX) [file pone.0332868.s001.docx]

**Appendix Table 1.** Description of the variables

|  | **Variables** | **Description** |
| --- | --- | --- |
| X1 | Floor type | The floor of the main dwelling is predominantly made of what material?  1 = Jute/plastic/others  2 = Mud  3 = Bamboo/wood  4 = Tin/CI sheet  5 = Concrete |
| X2 | Wall type | The outer walls of the main dwelling of the household are predominantly made of what material?  1 = Jute/plastic/others  2 = Mud  3 = Bamboo/wood  4 = Tin/CI sheet  5 = Concrete |
| X3 | Housing condition | What type of dwelling does the household live in?  1 = In a very poor state  2 = Very damaged  3 = Somewhat damaged  4 = Slightly damaged  5 = No sign of damage |
| X4 | Roof type | The roof of the main dwelling is predominantly made of what material?  1 = Jute/cardboard/grass/others  2 = Plastic/polythene/golpata  3 = Bamboo/wood  4 = Tin  5 = Concrete |
| X5 | Sanitation type | What type of latrine do you use?  1 = Open field/community latrine/other  2 = Kutcha (fixed place)  3 = Pucca(unsealed)  4 = Sanitary without flush  5 = Sanitary with flush |
| X6 | Source of water | What is the source of water used for drinking, cooking, taking a shower?  1 = Pond/River/ Canal/other  2 = Community tube well/ Ring Well  3 = Own tube well  4 = Supply Water (piped), outside  5 = Supply Water (piped) inside the house |
| X7 | Cooking fuel type | What is your main source of cooking fuel?  1 = Rice bran/ dried leaves/ others  2 = Firewood/dried cow dung/ coal  3 = LPG/Kerosene  4 = Supply gas  5 = Electricity |
| X8 | Health Distance | How long is the distance from your house to the closest facility?  1 = Very Far  2 = Far  3 = Moderate  4 = Near  5 = Closest |
| X9 | Bazar Distance | How long is the distance from your house to the closest facility?  1 = Very Far  2 = Far  3 = Moderate  4 = Near  5 = Closest |
| X10 | Employment location | Where is your employment location located?  1 = Another district  2 = Other than in this district  3 = Other union in this thana  4 = Other village/ward in this union  5 = This village/ward |
| X11 | Occupation type | What is your current main occupation type?  1 = Non-earning occupation  2 = Wage Labor  3 = Salaried worker  4 = Agricultural work  5 = Self-employment/ trader |
| X12 | Savings | How much money have you saved for the future?  1 = Very poor  2 = Poor  3 = Moderate  4 = High  5 = Very high |
| X13 | Income per month | How much money do you earn in a month?  1 = Very poor (<=10000 taka/month)  2 = Poor (>10000 & <=20000)  3 = Moderate (>20000 & <=30000)  4 = High (>30000 & <=50000)  5 = Very high (>50000 taka/month) |
| X14 | Household_head education year | Highest education class passed by the household head?  1 = Never attended school  2 = Primary level  3 = Secondary Level  4 = Higher Secondary Level  5 = Tertiary level |
| X15 | Diversify income | How much money comes from another source rather than the main source per year?  1 = Very poor  2 = Poor  3 = Moderate  4 = High  5 = Very high |
| X16 | Land size | Current size or area do you have?  1 = Very poor  2 = Poor  3 = Moderate  4 = High  5 = Very high |
| X17 | Land value | Current market value of the land?  1 = Very poor  2 = Poor  3 = Moderate  4 = High  5 = Very high |
| X18 | Livestock value | Current value/If livestock is sold today, how much will you receive?  1 = Very poor  2 = Poor  3 = Moderate  4 = High  5 = Very high |
| X19 | Livestock quantity | How much livestock do you have?  1 = Very poor  2 = Poor  3 = Moderate  4 = High  5 = Very high |
| X20 | Farm equipment value | Current value/If the asset is sold today, how much will you receive?  1 = Very poor  2 = Poor  3 = Moderate  4 = High  5 = Very high |
| X21 | Farm equipment quantity | How much asset do you have?  1 = Very poor  2 = Poor  3 = Moderate  4 = High  5 = Very high |
| X22 | Nonagri_asset value | How much asset do you have?  1 = Very poor  2 = Poor  3 = Moderate  4 = High  5 = Very high |
| X23 | Food consumption score | A weighted average is calculated on a household's weekly average consumption of various food categories.  1 = Very poor  2 = Poor  3 = Moderate  4 = High  5 = Very high |
| X24 | Food expenditure | A value, which includes purchased food for daily usage, is expressed in Bangladeshi Tk.  1 = Very poor  2 = Poor  3 = Moderate  4 = High  5 = Very high |

**Appendix Table 2.** Descriptive statistics

| Variables | Mean | Std. Dev. | Skewness | Kurtosis |
| --- | --- | --- | --- | --- |
| X1 | 2.736 | 1.289 | 1.183 | 2.406 |
| X2 | 3.951 | 0.938 | -1.127 | 3.946 |
| X3 | 3.603 | 0.872 | -0.067 | 2.6036 |
| X4 | 4.050 | 0.375 | -2.628 | 33.067 |
| X5 | 3.483 | 0.615 | -0.302 | 3.752 |
| X6 | 2.156 | 1.046 | -0.026 | 1.497 |
| X7 | 1.991 | 0.660 | 1.622 | 8.872 |
| X8 | 2.770 | 1.123 | 0.264 | 2.396 |
| X9 | 3.672 | 1.081 | -0.336 | 2.412 |
| X10 | 3.844 | 1.646 | -0.975 | 2.168 |
| X11 | 3.292 | 1.513 | -0.426 | 1.646 |
| X12 | 2.742 | 1.553 | 0.149 | 1.562 |
| X13 | 1.483 | 0.863 | 1.942 | 6.488 |
| X14 | 1.931 | 0.981 | 0.810 | 3.132 |
| X15 | 1.461 | 1.131 | 2.293 | 6.783 |
| X16 | 1.889 | 1.087 | 0.989 | 3.090 |
| X17 | 3.282 | 0.820 | 0.197 | 3.844 |
| X18 | 2.646 | 1.480 | 0.141 | 1.406 |
| X19 | 2.340 | 1.189 | 0.316 | 2.028 |
| X20 | 3.114 | 1.205 | -0.135 | 2.089 |
| X21 | 1.986 | 0.862 | 0.785 | 3.810 |
| X22 | 3.405 | 1.482 | -0.343 | 1.658 |
| X23 | 2.999 | 1.030 | 0.020 | 2.581 |
| X24 | 2.873 | 1.103 | 0.111 | 2.404 |
